# Carroll’s Literary Register

**Published:** 1865-07

**Authors:** 


### CARROLL'S LITERARY REGISTER.

We have before us the third number of this new semi-monthly, Published by R. W. Carroll & Co., booksellers of this city. The object of the work seems to be, to present notices and criticisms of recent works, so that the reader may have some just conception of a new work before purchasing. Such a work is very valuable to those who make it a point to be fully posted upon the literature of the day; indeed, this is a work which should be in the hands of every *reader*.

